# Diagnosis of mTBI in an ER Setting Using Eye-Tracking and Virtual Reality Technology: An Exploratory Study

**DOI:** 10.3390/brainsci15101051

**Published:** 2025-09-26

**Authors:** Felix Sikorski, Claas Güthoff, Ingo Schmehl, Witold Rogge, Jasper Frese, Arndt-Peter Schulz, Andreas Gonschorek

**Affiliations:** 1 Department of Neurotraumatolgy, BG Klinikum Hamburg, 21033 Hamburg, Germany; fsikorski@posteo.de; 2 Department of Clinical Research, BG Klinikum Unfallkrankenhaus Berlin, 12683 Berlin, Germany; claas.guethoff@ukb.de; 3 Department of Neurology, BG Klinikum Unfallkrankenhaus Berlin, 12683 Berlin, Germany; ingo.schmehl@ukb.de (I.S.); witold.rogge@ukb.de (W.R.); 4 Department of Trauma and Orthopedic Surgery, BG Klinikum Hamburg, 21033 Hamburg, Germany; j.frese@bgk-hamburg.de; 5 Department of Clinical Research, BG Klinikum Hamburg, 21033 Hamburg, Germany; a.schulz@bgk-hamburg.de; 6 Fraunhofer IMTE, 23562 Lübeck, Germany

**Keywords:** mTBI, virtual reality, eye-tracking, emergency department

## Abstract

**Background:** The aim of this study was to systematically explore point-of-care biomarkers as diagnostic indicators for the detection and exclusion of mild traumatic brain injury (mTBI) in an emergency room (ER) setting using Eye-Tracking and Virtual Reality (ET/VR) technology. The primary target group included patients who had suffered an acute trauma to the head and presented within 24 h to the emergency department. **Methods:** The BG Unfallkrankenhaus Berlin and the BG Klinikum Hamburg participated in this explorative, prospective, single-arm accuracy study. This study included patients who presented to the emergency department with suspected mTBI and were examined using ET/VR glasses. All further steps corresponded to clinical routine (e.g., decision on hospital admission, imaging diagnostics). After the completion of treatment, the patients were divided into mTBI and non-TBI subgroups by consensus between two independent clinical experts, who were blinded to the results of the index test (examination using ET/VR glasses) in the form of a clinical synopsis. The diagnosis was based on all clinical, neurological, neurofunctional, neuropsychological, and imaging findings. Routine trauma and neurological history, examination, and diagnosis were performed in each case. All statistical analyses were performed with exploratory intent. **Results:** The use of ET/VR glasses was found to be predominantly unproblematic. Two of the fifty-two analyzed parameters can be statistically distinguished from a random decision. No difference in oculomotor function was found between the two subgroups, and no correlations between the parameters recorded by the VR goggles and the detection of mTBI were found. **Conclusions:** At present, the use of VR goggles for the diagnosis of mTBI in an ER setting cannot be recommended.

## 1. Introduction

Mild traumatic brain injury (mTBI) is one of the most common neurological disorders, with an estimated annual incidence of 200–300/100,000 inhabitants [1]. It represents approximately 90% of all head trauma cases, making it the leading form of craniocerebral injury [2]. The main causes of mTBI are falls (42%), followed by traffic accidents (26%) and accidents at work or school (15%) [2]. In most cases, patients with suspected mTBI present to the emergency department (ED), where diagnosis and management are initiated [3]. 

The diagnosis of mTBI based on clinical criteria alone remains challenging. Studies have shown that up to 50% of patients sustaining an mTBI receive an inaccurate ED diagnosis [4,5]. Several factors contribute to this problem, such as memory lapses due to shock, stress reactions, or secondary injuries (e.g., hypotension), which may be misinterpreted as acute TBI. Clinical symptoms of mTBI are heterogeneous, including somatic complaints (headache, dizziness, nausea), cognitive impairments (attention, memory, executive function), and emotional disturbances (irritability, anxiety, depressed mood, sleep problems) [6]. These variables and often nonspecific symptoms complicate reliable assessment in the acute setting.

Accurate diagnostic classification has major therapeutic and prognostic consequences, influencing the need for inpatient admission, neuroimaging (CT/MRI), and decisions about return to work or sports participation [7]. Furthermore, recent research has shown that so-called “mild” brain injuries can result in significant mid- and long-term complications, including chronic post-traumatic syndrome [8], neural cellular alterations [9,10], chronic traumatic encephalopathy [11], and psychosocial consequences such as post-traumatic stress disorder and increased suicidality [12,13,14]. This contributes to increased healthcare utilization and further strains emergency departments, which are already operating at high capacity due to a growing number of head trauma cases [15,16]. Thus, there is a strong need for objective and accessible diagnostic methods for mTBI in the ED.

Oculomotor dysfunctions have been recognized as sensitive indicators of traumatic brain injury, since approximately half of all neuronal pathways are involved in the visual system [17,18,19,20,21,22,23,24,25,26]. Abnormalities are most commonly detected in saccades, anti-saccades, smooth pursuit, vergence, accommodation, and vestibulo-ocular reflexes. Several studies using video-oculography demonstrated that mTBI patients show measurable impairments in these domains, of ten correlating with white matter changes in MRI and with deficits in attention and working memory [27,28,29,30,31,32,33], These findings suggest that standardized oculomotor testing could provide an objective and clinically relevant marker of mTBI, although existing evidence is limited by small sample sizes and heterogeneous methodologies [34]. 

Virtual reality (VR)-based systems and portable eye-tracking (ET) devices now offer the possibility of standardized, point-of-care assessment of oculomotor function. Such technologies are transportable, easy to apply, and can detect neuropsychological and postural impairments after mTBI [35,36,37]. By integrating ET into VR goggles, it may become possible to objectively detect mTBI-related dysfunctions in the ED, a setting where diagnostic options are currently limited.

Therefore, the aim of this exploratory study was to evaluate the feasibility and diagnostic potential of VR-based eye-tracking for the early detection of mTBI in the emergency department. Our approach seeks to address the diagnostic gap in acute care by leveraging portable ET/VR technology as a novel tool for rapid and standardized assessment.

## 2. Patients and Methods

### 2.1. Patient Recruitment and Data Acquisition

Patients presenting to the ED within 24 h after an acute head trauma were screened for eligibility. mTBI was defined according to the American Congress of Rehabilitation Medicine criteria [38].

### 2.2. Inclusion Criteria

- Age 18–65 years
- Admission within 24 h after trauma
- Accident mechanism compatible with mTBI (e.g., direct head impact)

### 2.3. Exclusion Criteria

- Strabism;
- Pre-existing or traumatic damage to cranial nerves III (oculomotor nerve), IV (trochlear nerve) and VI (abducens nerve);
- Alcohol consumption (if suspected, confirmed by breath alcohol test);
- Sedating medication and intoxication;
- Multi-system injury;
- Seizures;
- Major physiological perturbations (e.g., hypotension).
- Moderate to severe TBI

In total, 122 patients fulfilled the inclusion criteria. After exclusion of 39 due to incomplete data, 82 patients remained for analysis. All data were pseudonymized. Baseline characteristics are summarized in Table 1.

### 2.4. Index-Test (ET/VR Examination)

Oculomotor function was assessed with EyeTrax VR-glasses (EyeTrax, Dual AMOLED 3.6, eyeTrax GmbH & Co. KG, Osnabrück, Germany). Oculomotor function was tested using a 3D simulation that triggered visual stimuli (see Appendix A and B for further information). The eye-tracking cameras recorded eye movements and pupil responses when tracking and searching for moving objects as well as light stimuli, using computer vision algorithms to detect and track the pupil in the video image. Due to the performance of the eye tracking used, EyeTrax was able to analyze fast, not consciously controllable eye movements, such as saccades, for both eyes.

This approach objectifies functional analysis and makes it largely independent of the subject’s motivation. Specific relevant eye movements can be triggered in a targeted and reproducible manner by using VR. Thus, the collected results can be compared on an interindividual basis under different circumstances (e.g., boxers before and after multiple head impacts) or across different subject groups. The development of these VR-based oculomotor protocols builds on prior work [39], which demonstrated the feasibility of immersive ET/VR systems for systematic assessment of eye movements in controlled environments.

The system enables short tests as well as the shielding of external stimuli and immersion, which significantly reduces external interference.

### 2.5. VR-Based Eye Tracking Protocols

A standardized sequence of tests was administered, covering saccades, smooth pursuit, vergence, vestibulo-ocular reflex, anti-saccades, and pupil responses. Details are provided in Table 2.

### 2.6. Reference Test

The clinical diagnosis of mTBI was made after completion of treatment and was blinded to the results of the index test (the ET/VR examination). The final classification (Mtbi Vs. Non was determined by consensus between two independent clinical experts based on a comprehensive clinical synopsis. The diagnosis incorporated all available findings, including clinical history, neurological and neurofunctional examination, neuropsychological assessment, and imaging results. In every case, a standard trauma and neurological assessment was performed according to routine clinical practice.

### 2.7. Statistical Analysis

The recorded videos were analyzed in several steps. First, an Ad Hoc pupil detection was carried out for the initial assessment to be able to evaluate the quality of the image.

In the second step, Post Hoc detection was carried out by eyeTrax, which enabled better data quality to be achieved, as several parameters could be determined, such as the sensitivity of the 3D eye model or contrast settings due to different video illuminations. The detection algorithm was tailored to the respective measurement in this step. The process took 5–10 min per measurement, depending on the length of the video.

The simulations were designed to record both eyes separately. Each partial movement (e.g., the ball shifting from left to right) was repeated ten times, depending on the specific simulation type.

To enable a high-level interpretation of the data with sufficient data quality, the various patterns within the eye tracking data had to be categorized. This annotation was performed manually, as automatic procedures resulted in too many false classifications in noisy data sets. Each data point was assigned to a class. The critical point here was to recognize when, for example, a saccade changes to a fixation, or when a blink is annotated, etc. This was carried out by trained staff with very good inter-operator reliability.

The classes obtained in this way could then be transferred to the various parameters and saved using automatic evaluations. The process was fully anonymized, i.e., at no time did an operator know which data set or test person was involved.

All statistical analyses were performed with exploratory intent. The primary aim was to investigate the suitability of the collected markers for the diagnosis of mild TBI in a consecutive cohort of patients exposed to external force application and to test the feasibility of the investigation by means of ET/VR.

All variables documented during the diagnostic routine were descriptively analyzed depending on their scale type. Absolute and percentage frequencies or mean and standard deviations or medians and ranges were used to describe the distributions.

For the exploratory analysis of markers, nonparametric receiver operating curves (ROCs) were calculated and the resulting area under the curves (AUCs) were first documented. Markers with AUC point estimates over 0.60 and a *p*-value of at least 0.05 when tested against the diagonal (AUC = 0.50) were analyzed regarding their mutual incremental diagnostic value and described using mean and standard deviations and ranges.

The subjects were divided into two groups (mTBI vs. non-mTBI). The parameters recorded were then compared between these two groups to see if there were any differences.

## 3. Results

The study cohort consisted of 82 patients (Table 1). Patients were divided into two groups (mTBI vs. non-mTBI) based on independent expert consensus.

### 3.1. SCAT3 Analysis

Analysis of the SCAT3 questionnaire revealed that the worsening of symptoms after mental and/or physical stress was particularly pronounced in patients with clinically confirmed mTBI. In contrast, the two groups showed relatively balanced results across most other SCAT3 domains (Table 3).

### 3.2. ET/VR Parameters

In total, 52 parameters were assessed using the ET/VR protocols. Exploratory ROC analysis revealed that two parameters reached statistical significance (*p* < 0.05 against the diagonal):

1. Velo Horizontal—number of eyelid closures (left/right merged)
2. Velo Horizontal—blink rate [#/s] (left/right merged)

All other ET/VR parameters did not show significant group differences (Table 4).

### 3.3. Numerical Group Comparisons

**Figure 1:**
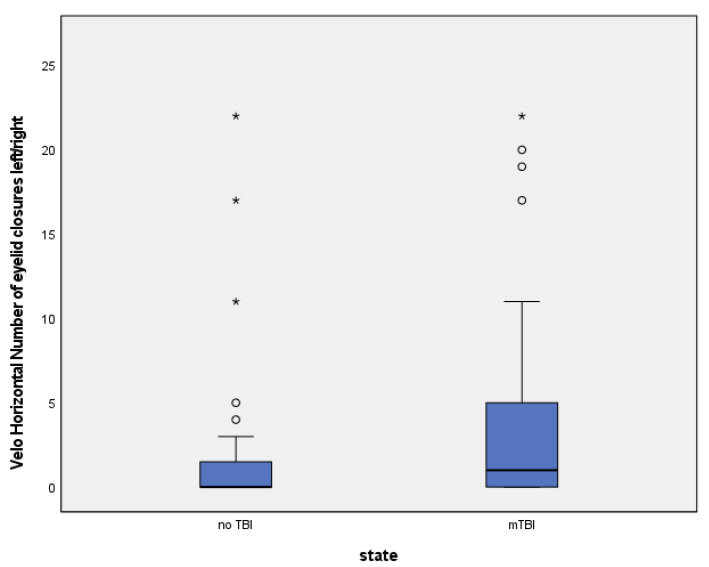
(Circles = outliers, stars = extreme values).

**Figure 2:**
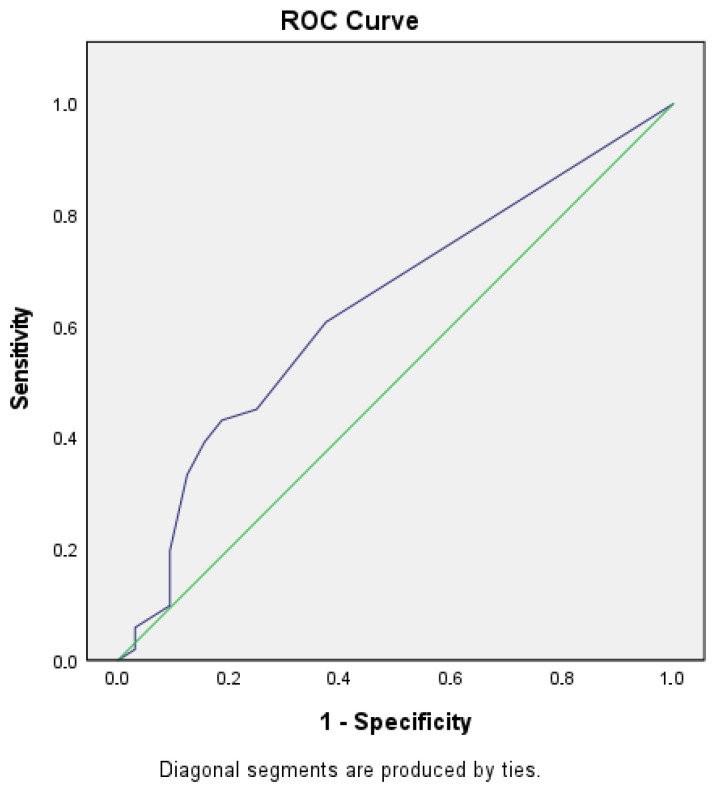
ROC 1.

**Figure 3:**
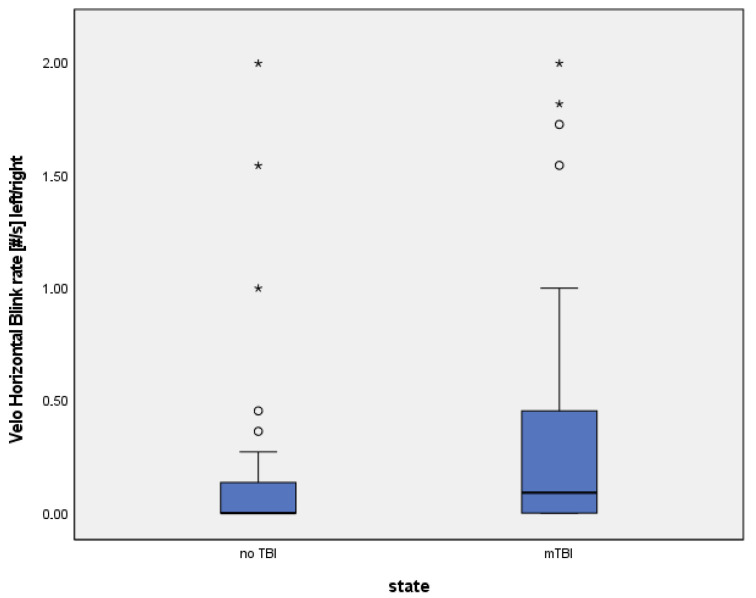
(Circles = outliers, stars = extreme values).

**Figure 4:**
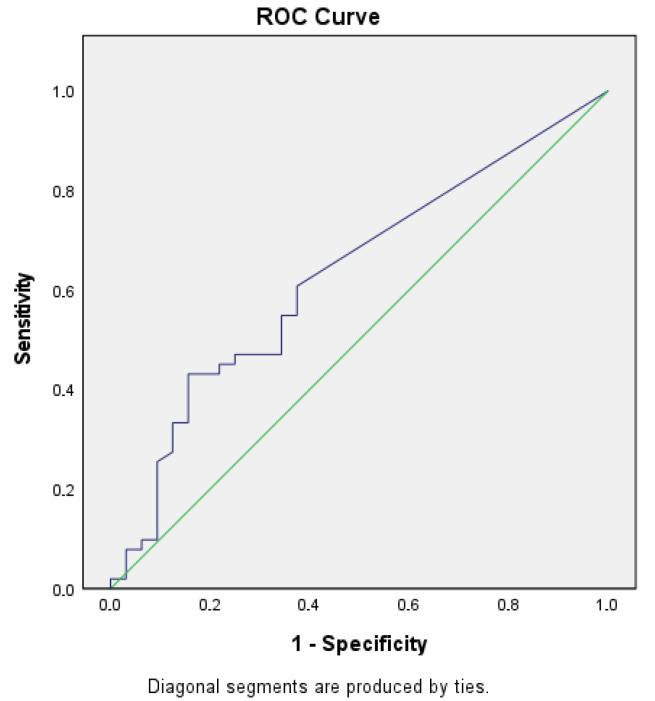
ROC 2.

- Number of eyelid closures: Patients with mTBI showed a higher number of eyelid closures during the Velo Horizontal task (median 7.5 vs. 5.0, *p* = 0.041). (Table 5, Figure 1 and Figure 2).
- Blink rate [#/s]: Median blink rate was higher in the mTBI group compared with non-mTBI controls (0.48 vs. 0.32 blinks/s, *p* = 0.037). (Table 6, Figure 3 and Figure 4).

Despite these differences, diagnostic accuracy remained moderate. The Velo Horizontal blink rate achieved an AUC of 0.63 (95% CI 0.51–0.75), with a sensitivity of 0.43 and specificity of 0.84 (Youden index 0.28). The number of eyelid closures yielded a similar AUC of 0.63 (95% CI 0.50–0.74), with a sensitivity of 0.43 and specificity of 0.81 (Youden index 0.24).

### 3.4. Physiological Implications

Increased blink rate and eyelid closure frequency may reflect disturbances in attentional control, fatigue regulation, and brainstem-mediated reflex circuits, which have been previously implicated in mTBI-related oculomotor dysfunction. However, due to the exploratory nature of this study and the modest effect sizes, these findings require cautious interpretation and further validation in larger cohorts.

### 3.5. β-Error Considerations

Given the sample size and the moderate AUC values, the risk of type II error (β-error) must be acknowledged. Subtle effects may not have been detected. This limitation is discussed in detail in the Section 4, with reference to prior work (e.g., [DOI: 10.1080/00913847.2018.1525261]).

### 3.6. Figures

To illustrate the distribution of blink rate and eyelid closures between groups, descriptives, boxplots and ROC (receiver operating characteristics) of blink reflex rate will be provided (Table 5 and Table 6, Figure 1, Figure 2, Figure 3 and Figure 4).

### 3.7. Side Effects and Practicability

Proportions of 10% of subjects from the mTBI and 6.3% from the non-TBI groups found the examination unpleasant. In total, 3 subjects (9.4%) from the mTBI and 7 (14.0%) from the non-TBI groups reported one minor side effect, like burning eyes, headache, neck pain, dizziness, and nausea. When asked whether they would have such an examination performed again, 87% of the mTBI and 92% of non-TBI groups answered yes.

### 3.8. Summary of Results

In summary, the SCAT3 revealed stress-related symptom worsening in mTBI patients, while ET/VR analysis identified two blink-related parameters with significant but moderate discriminatory power. These findings indicate potential oculomotor markers of mTBI, but their diagnostic value remains limited and requires further investigation. Patients reported few side effects and favorable practicability.

## 4. Discussion

The aim of this study was the systematic exploration of point-of-care biomarkers as diagnostic indicators for the detection and exclusion of mild traumatic brain injury (mTBI) in an emergency setting using Eye-tracking and Virtual Reality (ET/VR) technology. To our knowledge, this is the first study to investigate the feasibility of ET/VR technology in the ED for this purpose, thereby extending the application spectrum of this promising technology to clinical practice.

Existing diagnostic criteria for mTBI remain inconsistent, contributing to the challenges observed in our study. Heterogeneity in patient populations, assessment methodologies, and terminologies often leads to disparate classifications with limited clinical relevance. For example, a previous study [40] found that only 23% of patients meeting WHO criteria were clinically diagnosed with mTBI, suggesting underestimation in real-world settings. Similarly, broader concussion definitions based largely on self-reported symptoms further complicate the differentiation between concussion and mTBI [41]. This highlights the urgent need for standardized diagnostic tools.

### 4.1. Comparison with Current Diagnostic Methods

Current diagnostic practice in the ED primarily relies on clinical examination, patient-reported symptoms, and structural imaging (CT, MRI). While these methods are indispensable for detecting severe injuries and ruling out acute complications, they are often insufficient for diagnosing mTBI, since structural imaging is usually normal. Video-oculography (VOG) and neuropsychological testing have been explored, but they require specialized equipment, are sensitive to environmental disturbances, and are rarely feasible in the time-constrained ED setting.

To further contextualize ET/VR, it is useful to compare it with other established oculomotor screening tools. Unlike VOMS or the King-Devick test, which rely on symptom reporting and require baseline data, ET/VR provides objective, quantifiable oculomotor measures. This reduces susceptibility to patient motivation and environmental noise, making ET/VR particularly suitable for the emergency department setting.

### 4.2. ET/VR Technology Therefore Offers Several Potential Advantages

Portability and feasibility: VR-based ET can be applied directly at the bedside in the ED, without requiring transfer to specialized facilities.

Standardization: Immersive VR environments reduce external distractions and allow reproducible stimulus presentation across patients.

Objective quantification: Eye-tracking provides precise, automated measurements of oculomotor function that are less dependent on patient compliance.

Exploratory breadth: ET/VR enables testing of multiple functional domains (saccades, pursuit, vergence, blink reflexes) within short timeframes, supporting hypothesis-driven biomarker discovery.

At the same time, our study shows that the diagnostic performance of individual ET/VR parameters in the acute phase remains limited, with only moderate AUC values. These results underscore that ET/VR should not be viewed as a replacement for established methods but rather as a complementary tool with potential for further development.

### 4.3. Findings of the Present Study

In our exploratory analysis, two blink-related parameters (blink rate and eyelid closures during Velo Horizontal) showed significant but moderate discriminatory ability between mTBI and non-mTBI groups. Rather than indicating failure of the approach, these results underline the complexity of acute mTBI diagnostics. The findings suggest that oculomotor alterations may not be robustly detectable within the first 24 h after trauma, which was our study’s time window. This is consistent with prior research showing that abnormalities in oculomotor and visual tracking functions become more pronounced in the subacute phase (3–14 days post-injury) [29,42,43,44,45].

Several contextual factors may have influenced our results. The stressful and unpredictable ED environment can induce acute fatigue and stress reactions, which themselves affect oculomotor control and blink behavior [44,45,46]. In addition, elevated cortisol levels have been shown to alter network connectivity in the brain, including the Salience, Central Executive, and Default Mode Networks, all of which are linked to executive functions [45,46]. These mechanisms may have contributed to variability in our measurements.

## 5. Limitations

Our study has several important limitations. We focused on oculomotor biomarkers because they are sensitive to diffuse brain injury and can be measured non-invasively with ET/VR, but this excludes other relevant functional domains (sensory, cognitive, behavioral) that should be incorporated in future multimodal approaches. The study population was recruited exclusively from an emergency department, so recruiting healthy, non-injured controls was not feasible; this restricts interpretation of specificity and normative values, although it does reflect real-world conditions for an ED screening tool.

At the time of data collection the ET/VR system lacked integrated algorithms for automated eye-tracking analysis, requiring manual annotation of eyelid closures, fixations and saccades from synchronized video. While this improved quality control, it introduced potential observer bias; recent automated EyeTrax algorithms should reduce workload and variability in future work. The device could not be used in patients with scalp wounds for hygienic and safety reasons, so patients with external head trauma were excluded—limiting generalizability primarily to closed-head injuries.

Statistically, only 2 of 52 analyzed parameters reached *p* < 0.05 in the exploratory AUC analysis, and no α-adjustment was applied; therefore, type I error is possible. Conversely, moderate AUCs and a limited sample size raise the possibility of type II error and missed small effects. Taken together, these issues underscore the need for larger, prospective studies that include healthy controls, multimodal functional markers, serial post-trauma assessments, device refinements, standardized diagnostic protocols, and direct comparisons of ET/VR with conventional methods to establish clinical accuracy and utility.

### Strengths and Future Directions

Despite these limitations, our study demonstrates the feasibility of applying ET/VR technology in the challenging environment of an emergency department. Patient acceptance was high, testing was quick and well tolerated, and standardized oculomotor assessments could be performed at the bedside. These practical strengths underline the promise of ET/VR as a complementary diagnostic tool for mTBI.

Future research should integrate automated data analysis, include healthy control groups, and extend beyond oculomotor measures to encompass cognitive, sensory, and behavioral domains. Larger prospective trials, with repeated assessments at different time intervals post-trauma, will be needed to validate diagnostic accuracy and clarify the optimal time window for ET/VR testing. Ultimately, the integration of ET/VR with multimodal diagnostic approaches may contribute to earlier and more reliable detection of mTBI in clinical practice.

## 6. Conclusions

In conclusion, our study underscores the challenges of using exploratory eye-tracking and virtual reality (ET/VR) technology to identify oculomotoric changes associated with mild traumatic brain injury (mTBI) in an emergency department (ED) setting. Of the 52 parameters analyzed, only two showed statistically significant differences, indicating a moderate level of accuracy with an AUC of approximately 0.63. This limited success suggests that additional refinement and validation are needed to create reliable diagnostic tools for mTBI.

Several factors likely contributed to the modest results in our study. The absence of automated data processing within the ET/VR system created significant manual workloads and potential for human error. The inability to include patients with open skull injuries due to safety and hygiene concerns further narrowed our study’s scope, limiting the generalizability of our findings. Additionally, the lack of a healthy control group prevented robust comparison with typical eye movement patterns, which would be crucial in understanding the specific impact of mTBI.

Given the inconsistencies in mTBI diagnostic criteria and the difficulty in distinguishing mTBI from concussion, standardized methods and improved data processing in future ET/VR technologies are crucial. Further studies should aim to include a healthy control group, evaluate patients over a longer timeframe post-trauma, and ensure consistency in the diagnostic tools used. This will not only enhance the accuracy of mTBI detection but also contribute to a broader understanding of this condition’s clinical trajectory. The need for validated biomarkers and consistent terminology in mTBI research is clear and our study’s findings contribute to this ongoing discussion.

Despite these limitations, our study serves as an important step toward better diagnostic tools for mTBI and provides insights for future research directions. By addressing the identified limitations and building on our results, future research can more effectively harness ET/VR technology for mTBI diagnosis, leading to improved patient outcomes and a deeper understanding of brain injury dynamics.

## 7. Transparency, Rigor, and Reproducibility Summary

The study protocol was approved by the ethics committee of the Hamburg Medical Association (PV5863) and was registered with the German Clinical Trials Register (registration number: DRKS00032810), approval date 10/18/2018.

We conducted a multicenter, prospective, single-arm, exploratory clinical study to systematically explore point-of-care biomarkers as diagnostic indicators for the detection and exclusion of mild traumatic brain injury (mTBI) in an emergency setting using ET/VR technology.

This study was conducted at the BG Klinikum Hamburg and Unfallkrankenhaus Berlin. In the period from October 2019 to January 2022, 122 patients were included and examined, 58 of who were based in Hamburg and 64 in Berlin. Written informed consent was obtained from each participant prior to study participation.

The participants were not told the results of their prognostic assessments. Final clinical outcome assessments and adjudications were performed by team members blinded to the relevant characteristics of the participants. All surveys and questionnaires, as well as the examination protocols for the ET/VR glasses, are available from the authors. The key inclusion criteria and outcome evaluations are established standards.

## Figures and Tables

**Table 1:** Demographic and anamnestic patient characteristics with means (m) and standard deviations (StdDev.) for normal variables and absolute (No.) and relative (%) frequencies for dichotomous and polytomous variables.

		TBI State							
		No TBI				mTBI			
		Mean	Std. Dev.	No.	%	Mean	Std. Dev.	No.	%
Age (years)		41	15			39	15		
Sex	Male			16	51.6%			38	74.5%
	Female			15	48.4%			13	25.5%
History of head injuries (SHT)?	No			21	65.6%			25	49.0%
	Yes			11	34.4%			26	51.0%
Headaches (in general)	No			18	56.3%			25	49.0%
	Yes			14	43.8%			26	51.0%
Migraine	No			7	50.0%			17	65.4%
	Yes			7	50.0%			9	34.6%
Difficulty falling asleep	No			29	90.6%			45	88.2%
	Yes			3	9.4%			6	11.8%
Difficulty sleeping through the night	No			25	78.1%			46	90.2%
	Yes			7	21.9%			5	9.8%
Location of impact	Front			11	36.7%			17	40.5%
	Rear			10	33.3%			9	21.4%
	Right			7	23.3%			4	9.5%
	Left			0	0.0%			7	16.7%
	Top			2	6.7%			4	9.5%
	From below			0	0.0%			1	2.4%
Retrograde amnesia: Missing memories of events shortly before the injury?	No			30	93.8%			30	58.8%
	Yes			2	6.3%			21	41.2%
Anterograde amnesia: Missing memories of events shortly after the injury?	No			28	87.5%			27	54.0%
	Yes			4	12.5%			23	46.0%
Unconsciousness: Did the person concerned lose consciousness?	No			26	81.3%			26	52.0%
	Yes			6	18.8%			24	48.0%

**Table 2:** Overview of ET/VR protocols applied.

Test Name	Brief Description	Main Measurements	Clinical Relevance/Pathologies Detected
Biflicker (horizontal/vertical/3D)	Tracking of a moving orange ball across axes and in 3D	Saccadic amplitude, duration, velocity, acceleration	Detects deficits in basic saccadic control
Smooth pursuit (Sinus)	Ball moving along sinusoidal trajectories	Pursuit gain, intersaccadic interval	Identifies pursuit deficits, often impaired in mTBI
Diverge	Object increasing/decreasing in size while moving across the field	Amplitude, duration, velocity	Detects convergence/vergence dysfunction
Velo (horizontal/vertical)	Rapid shifts between two static targets	Amplitude, duration, velocity	Measures peak saccadic velocity, attention shifting
Anti-saccades	Suppress response to distractor stimulus	Latency, directional errors, accuracy	Sensitive to executive/attentional dysfunction in mTBI
Dice	Visual search of rotating 3D dice with numbers	Iris adaptation, saccadic control	Higher-order visual integration, fatigue detection
Flash	Light flashes are presented in series	Iris adaptation, pupil velocity	Photophobia, pupillary reflex alterations
Vestibular (horizontal/vertical)	Head/eye coordination with central fixation	Saccadic amplitude, duration, velocity	Vestibulo-ocular reflex assessment

**Table 3:** Variables of the SCAT 3 with 25th and 75th percentiles and median (md), absolute (No.), and relative (%) frequencies.

		TBI State									
		No TBI					mTBI				
		25th Perc	md	75th Perc	No	%	25th Perc	md	75th Perc	No	%
Headache		0	2	3			1	2	3		
“Pressure in head”		0	1	2			0	2	3		
Neck Pain		0	1	3			0	2	3		
Nausea or vomiting		0	0	1			0	0	1		
Dizziness		0	0	2			0	1	2		
Blurred vision		0	0	0			0	0	1		
Balance problems		0	0	0			0	0	1		
Sensitivity to light		0	0	1			0	0	1		
Sensitivity to noise		0	0	0			0	0	0		
Feeling slowed down		0	0	1			0	0	2		
Feeling like “in a fog”		0	0	1			0	1	2		
“Don’t feel right”		0	0	1			0	1	2		
Difficulty concentrating		0	0	1			0	0	1		
Difficulty remembering		0	0	0			0	0	2		
Fatigue or low energy		0	1	3			1	2	3		
Confusion		0	0	0			0	0	0		
Drowsiness		0	0	1			0	0	2		
Trouble falling asleep		0	0	0			0	0	0		
More emotional		0	0	0			0	0	1		
Irritability		0	0	0			0	0	0		
Sadness		0	0	0			0	0	1		
Nervous or Anxious		0	0	0			0	0	0		
Do the symptoms get worse with physical or mental activity?	No				25	83.3%				32	66.7%
	Yes				5	16.7%				16	33.3%

**Table 4:** AUCs including standard errors, *p*-values, and limits of the 95% confidence interval of the parameters recorded using ET/VR glasses.

	AUC	Standard Error	*p*	Lower Limit with 95% CI	Upper Limit with 95% CI
Velo Horizontal Number of eyelid closures left/right	0.634	0.062	0.041	0.511	0.756
Velo Horizontal Blink rate [#/s] left/right	0.630	0.063	0.048	0.507	0.752
Biflicker Main saccade amplitude [°] right/Biflicker Gain of main saccade [%] right	0.612	0.064	0.089	0.486	0.737
Velo vertical average peak velocity [°/s] left	0.400	0.067	0.125	0.269	0.530
Biflicker Vertical V_max of the main saccade [°/s] left	0.404	0.065	0.142	0.277	0.531
Biflicker Duration of the main acceleration [ms] left	0.405	0.062	0.148	0.283	0.527
Biflicker direction with respect to the *X*-axis in the UZS. [°] right	0.589	0.065	0.172	0.462	0.717
Velo vertical Number of saccades right	0.585	0.064	0.197	0.459	0.710
Velo vertical Number of saccades left	0.584	0.064	0.202	0.458	0.710
Velo Horizontal Average peak speed [°/s] left	0.424	0.068	0.244	0.291	0.557
Biflicker Vertical trigger direction with regard to the *X*-axis in the UZS. [°]	0.571	0.065	0.278	0.444	0.698
Biflicker V_max of the main saccade [°/s] left	0.431	0.064	0.295	0.305	0.557
Biflicker Vertical Blink rate [#/s] left/right	0.567	0.063	0.303	0.444	0.691
Velo Horizontal Duration of repetition [ms]	0.566	0.066	0.310	0.438	0.695
Biflicker Vertical Number of eyelid closures left/right	0.564	0.063	0.331	0.440	0.687
Biflicker Vertical Number of saccades left/right	0.437	0.064	0.335	0.311	0.563
Biflicker Duration of repetition [ms]	0.561	0.065	0.352	0.433	0.689
Velo Horizontal Average precision error [°] right	0.444	0.063	0.392	0.320	0.568
Velo Horizontal Average peak speed [°/s] right	0.447	0.066	0.416	0.318	0.575
Biflicker Vertical Duration of repetition [ms]	0.451	0.066	0.454	0.322	0.580
Biflicker Main saccade amplitude [°] left/Biflicker Gain of the main saccade [%] left	0.547	0.065	0.471	0.420	0.674
Velo Horizontal Average precision error [°] left	0.454	0.064	0.486	0.329	0.580
Velo Vertical Duration of repetition [ms]	0.456	0.065	0.507	0.330	0.583
Biflicker direction with respect to the *X*-axis in the UZS. [°] left	0.538	0.066	0.562	0.409	0.67
Biflicker Number of saccades left	0.533	0.064	0.610	0.408	0.659
Biflicker Vertical V_max of the main saccade [°/s] right	0.467	0.069	0.617	0.332	0.603
Biflicker vertical Duration of main saccade [ms] left/right	0.470	0.067	0.650	0.339	0.602
Biflicker vertical direction with respect to the *X*-axis in the UZS. [°] right	0.527	0.065	0.677	0.399	0.655
Biflicker Latency of the main saccade to the trigger [ms] left	0.474	0.065	0.694	0.346	0.602
Biflicker vertical gain of the main saccade [%] right	0.475	0.067	0.698	0.343	0.606
Biflicker Vertical gain of main saccade [%] left	0.477	0.066	0.722	0.347	0.607
Biflicker Number of saccades right	0.522	0.064	0.733	0.397	0.648
Biflicker vertical main saccade amplitude [°] left	0.478	0.066	0.736	0.349	0.607
Velo vertical blink rate [#/s] left	0.521	0.065	0.747	0.394	0.649
Velo vertical blink rate [#/s] right	0.521	0.065	0.747	0.394	0.649
Biflicker Vertical Main saccade amplitude [°] right	0.479	0.068	0.754	0.347	0.612
Velo Vertical Number of eyelid closures left/right	0.521	0.065	0.754	0.393	0.648
Biflicker Vertical Latency main saccade to trigger [ms] left/right	0.519	0.065	0.775	0.390	0.647
Biflicker directional error prosaccade [%] right	0.517	0.067	0.793	0.386	0.649
Biflicker Vertical Direction error prosaccade [%] left	0.488	0.068	0.859	0.355	0.622
Velo Vertical Average precision error [°] left	0.490	0.064	0.881	0.364	0.616
Biflicker Vertical Direction with respect to the *X*-axis in UZS. [°] left	0.509	0.065	0.896	0.381	0.636
Biflicker V_max of the main saccade [°/s] right	0.508	0.069	0.899	0.373	0.644
Biflicker trigger direction with respect to the *X*-axis in the UZS. [°]	0.508	0.065	0.903	0.380	0.636
Velo Horizontal Number of saccades left	0.493	0.066	0.918	0.365	0.622
Biflicker direction error prosaccade [%] left	0.494	0.068	0.925	0.361	0.626
Velo Horizontal Number of saccades right	0.494	0.066	0.925	0.365	0.622
Biflicker Number of left/right eyelid closures	0.506	0.065	0.925	0.378	0.634
Velo Vertical Average precision error [°] right	0.505	0.069	0.944	0.369	0.640
Biflicker Blink rate [#/s] left/right	0.504	0.065	0.948	0.376	0.633
Biflicker Vertical Directional error prosaccade [%] right	0.498	0.067	0.978	0.366	0.630
Velo Vertical Average top speed [°/s] right	0.502	0.066	0.978	0.373	0.630

**Table 5:** Velo Horizontal Number of eyelid closures left/right Velo Horizontal Number of eyelid closures left/right.

Descriptives					
	State			Statistic	Std. Error
Velo Horizontal Number of eyelid closures left/right	no TBI	Mean		2.19	0.900
		95% Confidence Interval for Mean	Lower Bound	0.35	
			Upper Bound	4.02	
		5% Trimmed Mean		1.31	
		Median		0.00	
		Variance		25.899	
		Std. Deviation		5.089	
		Minimum		0	
		Maximum		22	
		Range		22	
		Interquartile Range		2	
		Skewness		3.008	0.414
		Kurtosis		8.874	0.809
	mTBI	Mean		3.71	0.765
		95% Confidence Interval for Mean	Lower Bound	2.17	
			Upper Bound	5.24	
		5% Trimmed Mean		2.97	
		Median		1.00	
		Variance		29.812	
		Std. Deviation		5.460	
		Minimum		0	
		Maximum		22	
		Range		22	
		Interquartile Range		5	
		Skewness		2.040	0.333
		Kurtosis		3.846	0.656

**Table 6:** Velo Horizontal Blink rate [#/s] left/right.

Descriptives					
	State			Statistic	Std. Error
Velo Horizontal Blink rate [#/s] left/right	no TBI	Mean		0.1985	0.08162
		95% Confidence Interval for Mean	Lower Bound	0.0320	
			Upper Bound	0.3650	
		5% Trimmed Mean		0.1191	
		Median		0.0000	
		Variance		0.213	
		Std. Deviation		0.46171	
		Minimum		0.00	
		Maximum		2.00	
		Range		2.00	
		Interquartile Range		0.16	
		Skewness		3.008	0.414
		Kurtosis		8.872	0.809
	mTBI	Mean		0.3364	0.06940
		95% Confidence Interval for Mean	Lower Bound	0.1970	
			Upper Bound	0.4758	
		5% Trimmed Mean		0.2701	
		Median		0.0908	
		Variance		0.246	
		Std. Deviation		0.49563	
		Minimum		0.00	
		Maximum		2.00	
		Range		2.00	
		Interquartile Range		0.45	
		Skewness		2.040	0.333
		Kurtosis		3.843	0.656

## Data Availability

The raw data supporting the conclusions of this article will be made available by the authors on request.

## Appendix A

Technical Specifications

## Appendix B

Video of the simulation.
